# P-37. Risk Factors for Disseminated Viridans Streptococcal Bacteremia: Impact of Oral Health Among Older Adults

**DOI:** 10.1093/ofid/ofaf695.266

**Published:** 2026-01-11

**Authors:** Kota Minami, Takashi Matono, Yusuke Oka, Toshiharu Urakami, Yosuke Aoki

**Affiliations:** Saga University Hospital, Saga, Saga, Japan; Saga University Hospital, Saga, Saga, Japan; Saga University, Saga city, Saga, Japan; Saga University Hospital, Saga, Saga, Japan; Saga University, Saga city, Saga, Japan

## Abstract

**Background:**

Poor oral health can cause localized odontogenic infections, which may progress to surrounding tissues and even cause severe systemic infections. However, there is limited research on the risk factors for development of disseminated infections caused by viridans streptococci, which could pose a serious concern, particularly in aging populations.

**Figure:**
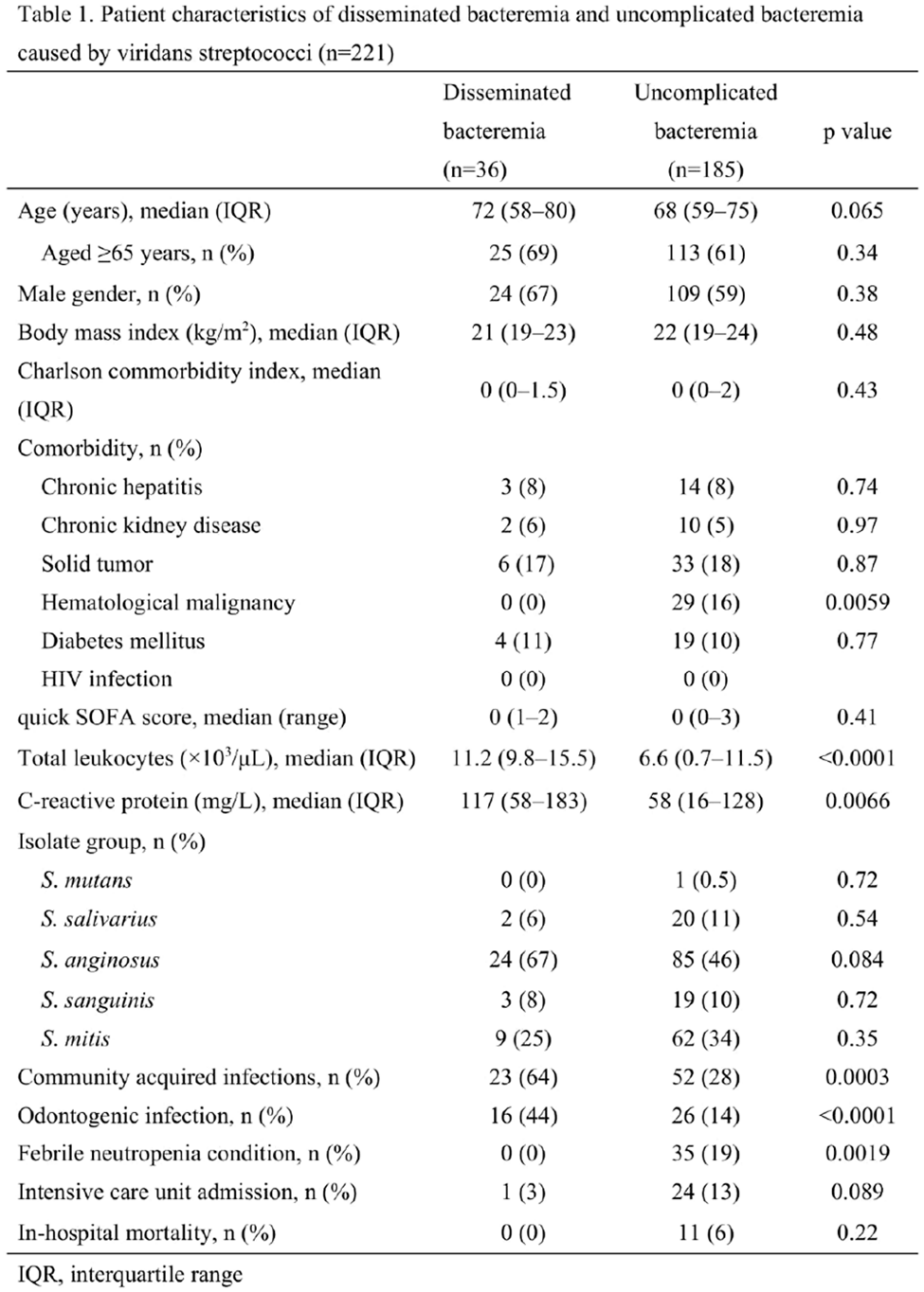

Site of infections in patients with disseminated bacteremia (n=36)

**Figure:**
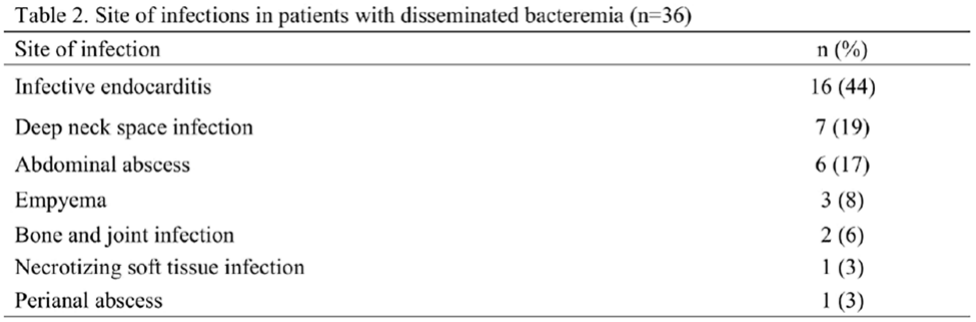

**Methods:**

This retrospective study included patients aged 18 years and older with viridans streptococci bacteremia who were admitted to Saga University Hospital in Japan between 2014 and 2024. Cases involving polymicrobial bacteremia, patients presenting with cardiopulmonary arrest upon arrival, and instances considered to be contamination were excluded.

**Figure:**
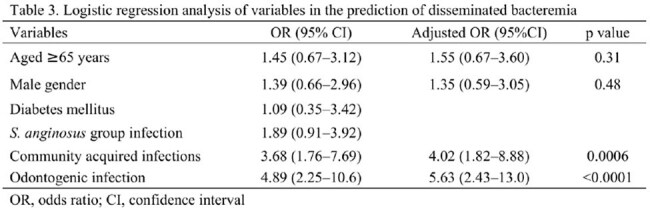
Logistic regression analysis of variables in the prediction of disseminated bacteremia

**Results:**

A total of 221 patients were analyzed, including 36 with disseminated bacteremia and 185 with uncomplicated bacteremia. The most frequently identified isolate was the *S. anginosus* group (49%), followed by *S. mitis* group (32%). Among patients with disseminated bacteremia, the most common sites of infection were infective endocarditis (44%), deep neck space infections (19%), and abdominal abscesses (17%). The median age (72 vs 68 years, p=0.065) and Charlson Comorbidity Index (p=0.43) were similar between the two groups. Multivariate logistic regression analysis revealed that community-acquired infections and odontogenic infections were significantly associated with disseminated bacteremia, with adjusted odds ratios of 4.02 (95% CI: 1.82–8.88) and 5.63 (95% CI: 2.43–13.0), respectively.

**Conclusion:**

These findings highlight the potential clinical impact of oral health and the source of infection on the severity of bacteremia. This study suggests the importance of early preventive interventions for maintaining oral health in older adults. Healthcare professionals should recognize that proactive management and preventive oral care strategies are essential for reducing the risk of potentially life-threatening infections.

**Disclosures:**

Takashi Matono, MD, PhD, FACP, Gilead Sciences: Honoraria|GSK: Honoraria|Meiji Seika Pharma: Honoraria|MSD: Honoraria|Pfizer: Honoraria

